# Implementation of open dialogue in Germany: Efforts, challenges, and obstacles

**DOI:** 10.3389/fpsyg.2022.1072719

**Published:** 2023-02-09

**Authors:** Kolja Heumann, Mira Kuhlmann, Maike Böning, Helene Tülsner, Raffaella Pocobello, Yuriy Ignatyev, Volkmar Aderhold, Sebastian von Peter

**Affiliations:** ^1^Integrated Working Group “Mental Health”, Brandenburg Medical School, Brandenburg an der Havel, Germany; ^2^Department of Psychology, University of Marburg, Marburg, Germany; ^3^Department of Social Work, Alice Salomon University, Berlin, Germany; ^4^National Research Council of Italy, Institute of Cognitive Sciences and Technologies, Rome, Italy; ^5^Department of Psychiatry and Psychotherapy, Immanuel Hospital Rüdersdorf, Brandenburg Medical School, Rüdersdorf, Germany; ^6^Department of Psychiatry, Charité University Medicine, Berlin, Germany

**Keywords:** Open Dialogue approach, implementation, mental health care, crisis, Expert interviews, need-adapted, fidelity

## Abstract

**Purpose:**

The Open Dialogue (OD) approach has been implemented in different countries worldwide. OD not only depends on therapeutic principles but also requires a distinct set of structural changes that may impede its full implementation. In Germany, OD is currently practiced in different mental health care settings across the country. Yet, full implementation of OD principles is limited due to the extreme structural and financial fragmentation of the German mental health care system. With this as a background, the aim of this study was to investigate the efforts, challenges and obstacles of OD implementation in Germany.

**Methods:**

This article presents the German results from the international HOPEnDIALOGUE survey, supplemented with expert interview data. Thirty eight teams currently providing OD took part in the survey. Sixteen expert interviews were carried out with stakeholders from various care settings. Survey data were analyzed descriptively and the qualitative data were evaluated using a thematic analysis approach.

**Results:**

While having to adapt to the fragmented German health care system, OD has been mainly implemented from outpatient service providers and stand-alone services. About half of the teams implemented OD under the conditions of cross-sectoral model contracts and, thus, are considerably limited when it comes to OD implementation. Altogether, OD is not implemented to its full extent in each of the institutions surveyed. Similarly, the expert interviews revealed various challenges that mainly relate to the realization of OD’s structural principles, whereas the implementation of its therapeutic benefits remains less affected. However, these challenges have managed to lead to great commitment by single teams and a certain level of implementation of OD-related concepts.

**Conclusion:**

OD in Germany can currently only be fully implemented under the cross-sectoral care model contract system that is often temporary, thus significantly hindering its continuous development. Any evaluation of OD’s effectiveness in Germany thus needs to take into account the fragmented nature of the country’s health care system and control for the multiple barriers that impede implementation. Reforms of the German health care system are also urgently needed to create more favorable conditions for the implementation of OD.

## Introduction

Open Dialogue (OD) is an integrated approach of continuous community-based and multiprofessional psychiatric support in severe crises, originally developed in Western Lapland, which involves the social network of service users from the very beginning. The central (therapeutic) element are network meetings with the users and their environment with the aim of promoting dialogue in the network, enabling mutual understanding of the respective perspectives on the current situation, and empowering clients and their networks to make joint decisions for further actions and desired changes. All other therapy services, e.g., individual psychotherapy, social work, etc., are provided and integrated as needed. In the original model, this explicitly also refers to the handling of medication, which is often only used selectively and after joint consultation due to the safeguarding framework of joint network meetings (Seikkula et al., 2006). Accordingly, OD is seen as an important and promising approach to a more person- and recovery-oriented and rights-based mental health care (Von Peter et al., 2019, 2021; WHO, 2021).

The OD model essentially consists of a community-based treatment structure as well as a specific dialogic conversation approach and is often described in terms of seven basic principles (Aaltonen et al., 2011; Seikkula et al., 2011): 1. Immediate help in crises, ideally within 24 h; 2. Involvement of the social network through network meetings from the beginning of the treatment; 3. Flexibility and mobility with regards to the needs of the network in terms of frequency, location and participants in network meetings; 4. Responsibility for the organization and implementation of the entire treatment process by the treatment team; 5. Psychological continuity or ensuring the continuity of relationships and common understandings over the entire course of treatment; 6. Tolerating uncertainty during network meetings and the entire treatment process and 7. Promoting dialogue and polyphony between network members as well as the staff members.

Since its development, different cohort studies have shown promising results in clinical, economic and social outcomes (Seikkula et al., 2006; Aaltonen et al., 2011; Seikkula et al., 2011; Bergstrøm et al., 2017; Bergström et al., 2018). This has led to the recognition and dissemination of OD worldwide (Buus et al., 2021). Currently, OD has been adapted and at least partially implemented in 25 countries (Pocobello et al., 2022), although it should be noted that the original practice of progressively withholding neuroleptic treatment for people experiencing first-episode psychoses has been implemented so far only in Western-Lapland and nowhere else in this world. Concerning the implementation, various obstacles have been described, related both to OD structural and therapeutic principles (Buus et al., 2021). A recent interview study mentioned, for example, that OD is not a “manualized” treatment method, explaining why this may generate tensions in organizational implementation that “favor specific and standardized practices” (Lennon et al., 2022). In another ethnographic study, the core values of OD are described as being in conflict with the “expectations of professional practices and performance” (Dawson et al., 2019). Moreover, it has been reported that adopting OD, like any other organizational change, may generate “organizational, professional, and personal resistance” (Søndergaard, 2009).

Thus, and in spite of this, OD may be adapted to different contexts (Seikkula and Arnkil, 2006) and should be adjusted to local conditions, while knowing that its implementation will encounter various obstacles and challenges. This stems from the fact that the OD approach is not only a particular intervention or a specific form of therapeutic conversation, but also requires certain changes on the structural and organizational levels to be implemented (Seikkula and Arnkil, 2006; Haarakangas et al., 2007). Thus, the success of its implementation depends on the specific conditions of the contextualizing health care systems, strongly affecting the realization of its structural and therapeutic principles (Seikkula et al., 2001), which are often adapted to suit local conditions (Buus et al., 2021). For these reasons, the following section presents some facts and structural details of the German mental health system that are needed to understand the subsequent results of our study. The main research question, which obstacles and challenges hinder the implementation of OD to its full extent makes it necessary to address some of the financial and structural specifics of the German mental health care situation.

### 1.1. OD in the German mental health system

In Germany, OD has met with a great response and many providers of (community) psychiatric care have had their staff trained further and have also oriented their range of services to include OD (Steinert et al., 2020; Von Peter et al., 2020). Since 2007, 77 Open Dialogue trainings have been conducted with about 30 participants each, mostly made possible through the commitment of one of the authors (VA). In close cooperation with the training groups, a curriculum has been developed (Aderhold and Borst, 2016), resulting in a training program over 16 days (8 workshops) that is compatible in terms of time and economy for providers of psychosocial care. Besides theoretical inputs, it is largely focused on staged exercises and self-experience for each key element and full-length coached role plays of network meetings through identification with own clients. With the aim of networking among different service providers, the workshops were either conducted with participants from several providers in a region or as in-house training, often with participants from other regional cooperation partners, mostly several courses in a row. In addition, course graduates were increasingly included as co-trainers after they had gained sufficient practical experience in conducting network meetings. In this way, more regional co-trainers were soon available, and regional trainer networks have since been formed for further training.

However, despite this great commitment, a full implementation, especially of OD’s structural principles remains limited due to the structural and financial fragmentation of the German health care system. Currently, routine psychiatric care financed by health insurance schemes is mainly provided by psychiatric inpatient or outpatient clinics and office-based psychiatrists or psychologists. These are supplemented by a broad spectrum of nonmedical, residential, occupational, rehabilitative and other psychosocial services (Salize et al., 2007), financed by other cost units like pension funds or taxes. In the absence of a comprehensive national policy on mental health, this fragmentation of service providers and payers leads to large regional disparities in the variety of offer, content and quality of services (Bramesfeld et al., 2012) and, even within one region, services are often poorly integrated. In particular, the transition between inpatient and outpatient care is often not well coordinated and the risk of discontinuity of care during this transition process is quite high (Puschner et al., 2012). Accordingly, intensive outreach community mental health care programs (e. g. ACT, CRT) have rarely been implemented in the German psychiatric care system despite the national and international evidence of their effectiveness (Gühne et al., 2018; Von Peter et al., 2019), except from some innovative financing frameworks that have emerged within the past few years. Since 2013, so-called “model projects” (§64b, SGB V) have made it possible to further develop hospital-based yet cross-sectoral mental health care approaches, leading to more needs-based care and support and a diversity of approaches with home treatment (Bauer et al., 2016). Moreover, since 2007 the so-called “integrated care” according to §140a (SGB V) offers the opportunity to integrate different service sectors and interprofessional treatment groups, thus improving the continuity and quality of care mainly in the outpatient sector (Schwarz et al., 2020). Both projects use *model contracts* apart from the regular funding system for testing new forms of service structures and thus are limited either in scope to certain users or health insurance companies, as well as in terms of treatment and contract duration.

Given this context, there is currently no regular funding that better enable a person-centered, needs-based, and cross-sectoral mental health care approach and thus allowing for more comprehensive implementation of OD (Von Peter et al., 2020). Thus, an OD-related support could only be provided under the limiting conditions of standard care or in the context of limited model contracts. Accordingly, the first trainings were conducted only in services without regular funding (e.g., integrated care). As this practice became better known, trainings in regularly funded structures were added, in the hope of slowly ‘eroding’ the overall system and knowing that only a partial implementation would be possible. And in terms of concrete treatment pathways, this means that patients can only receive OD-oriented treatment at selected facilities. Within regular care, largely in clinics without the capacity for early outpatient care and limited to a short treatment period. And within model projects, often for longer periods, but limited to individual health insurers and not for clients in acute crises.

### 1.2. Aim of the study

Against this background, it is of great interest to uncover to what extent the OD approach can currently be implemented in Germany. The aim of the present study was to evaluate the status of implementation practices and fidelity to the OD principles. In addition, the specific challenges and obstacles that make OD implementation and realization difficult under the current conditions of the German mental health care system were investigated in more detail.

## 2. Materials and methods

This article presents part of the results of the HOPEnDIALOGUE online survey [the procedures and main results will be presented in a dedicated article (Pocobello et al., 2022)], an international collaborative multicenter study investigating the implementation and effectiveness of Open Dialogue in different countries/contexts (www.hopendialogue.net). The survey was completed by expert interviews on the specific efforts, challenges and obstacles of implementing and practicing OD within the German mental health care system. This allowed us to use a two-fold research approach, with the survey giving an overview of implementation status in Germany and expert interviews providing for an in-depth understanding of the related problems and challenges. The online survey was conducted from February to September 2020, followed by expert interviews from September to November 2020.

### 2.1. Materials

As part of the first phase of the HOPEnDIALOGUE project, the online survey was conducted to emphasize the number of OD-providing services worldwide and the extent of implementation. For this purpose, a questionnaire consisting of 65 items was developed by the HOPEnDialogue research group, covering different topics (e.g., general information on the facility, information on the OD service provided and level of fidelity of OD) (Pocobello et al., 2022). Most of the questions included single-, multiple-choice and free-text responses. Fidelity to the principles of OD was measured with 18 items on a 5-point Likert scale (“never” – “almost always”), each of which was phrased as a statement about compliance with an OD-principle to provide a service (e.g., “The first meeting takes place within 24 h after the request for help.”). We translated the original version for the German survey and added 12 further questions that specifically related to the German context (e.g., the financial framework conditions and the reasons for implementation). The survey was freely accessible and self-administrated using UniPark EFS Survey (Tivian XI GmbH, Cologne, Germany).

The expert interviews were conducted to gain a deeper understanding of what helps and what impedes the implementation and practice of OD within the German health care system. The interview guide was developed in four stages according to Helfferich (Helfferich, 2011). First, some of the authors (MB, HT, SvP) developed the questions on the object of the study, incorporating existing knowledge of OD and based on other models of health services research (Proctor et al., 2009; Brooks et al., 2011). These questions were then checked for fit and duplication and discussed with the rest of the research team (KH, MK, VA). The questions were then sorted and clustered into thematic interview sections, and a guiding question was formulated for each of these sections. In this way, the finalized guide emerged covering three thematic sections: 1. implementation process and implementation practice in the respective institutions, 2. comparison between the model and the actual implementation and 3. interests and hopes for maintaining and further developing the OD services offered. Using the format of an open interview, a theory-practice comparison was also discussed using a list of OD principles. The full interview questionnaire can be found in electronic annex to this publication (Supplementary Table 1).

### 2.2. Recruitment

The recruitment for both the qualitative and standardized assessments was done in collaboration with the Open Dialogue Network in Germany, a self-organized association of professionals and interested parties who practice Open Dialogue, offer training and/or are committed to the practice’s dissemination in Germany (Von Peter et al., 2020). Through this network, we had a fairly reliable overview of all institutions in Germany offering OD in their services. This allowed us to aim for a full survey of 43 inpatient and outpatient facilities that, to our knowledge, were offering OD-inspired services at the time of the study. Teams with individually trained staff members but where OD practices were not a relevant part of services offered were not included.

To recruit for the online survey, the study was advertised in advance during the bi-annual meetings of the network and a list of all potential facilities was compiled. When the study began, an invitation to participate was sent, asking for completion of the survey by a team member with leadership responsibility on behalf of the entire facility or OD-Team. Reminders to participate were sent again at 3- and 6-week intervals.

Participants for the expert interviews were also recruited as a subsample of the survey participants with the greatest possible heterogeneity in terms of age, gender, profession, professional experience, and the settings/conditions in which they practice OD. Staff from institutions where the survey results had showed extensive implementation practices were included, as well as staff working in institutions with rather low levels of OD implementation. The interviews lasted an average of 90 min (range: 55–148 min.).

**Table 2:** Characteristics of respondents interviewed (*n* = 16).

		*n*	Ø	SD
Total		16		
Female		11		
Age (years)			49.56	10.85
Work experience (years)			21.06	10.89
Profession	Nurse	2		
	Social work/education	6		
	Psychology	4		
	Medicine	3		
Type of Service^1^	Outpatient crisis service	7		
	Residential Care	6		
	(day) Hospital	5		
Legal framework^1^ (funding)	Cross-sectoral model contract (§64b, § 140a)^2^	9		
	Standard care contract	9		

1 multiple answers possible.

2 model contracts via “model projects” according to §64b, SGB V and “integrated care contracts” according to §140a, SGB V.

### 2.3. Analysis

Survey data were mostly evaluated descriptively. The characteristics of the facilities partaking were summarized in absolute and relative frequencies. The degree of implementation based on the fidelity items was illustrated in relative frequencies depending on the different response options. An exploratory analysis of potential differences in the various fidelity items between facilities with different service focus and funding was conducted non-parametrically using Mann–Whitney-U-Tests. The significance level was set at 5% (two-sided).

The qualitative data from the expert interviews were evaluated using a thematic analysis approach and using MAXQDA Software (VERBI Software, Berlin, Germany). Categories were formed both inductively based on the interviewees’ response material, and deductively in light of OD principles (Seikkula et al., 2001). Each transcript was coded twice (by HT and MB) to increase reliability. Codes were discussed and agreed upon in a subgroup of the research team (HT, MB, SvP). For the purpose of this publication, the code tree was revised and adapted (by KH) to select suitable material that reflects the survey data. As a result, not all categories are reported in this article. The complete category system is available from the authors upon request.

## 3. Results

The survey results are presented first, followed by data from the expert interviews that provide detailed insight into the specific challenges and obstacles to implementing OD in Germany.

### 3.1. Survey data

Out of a total of 43 facilities and teams practicing OD which were contacted, 41 from almost all of the federal states participated in the survey (response rate: 76.7%). If more than one team from one and the same institution were practicing OD under different framework conditions, they took part as separate teams in the survey, which occurred in 6 cases. Due to incompletely filled out questionnaires the data of three OD-teams had to be excluded. In the end, this resulted in 38 data sets of teams that were included in the evaluative analysis. Although the overall survey data was extensive, only the data on the implementation of and fidelity to the OD principles that fit the research questions are presented below. First, the characteristics of the OD institutions are described in more detail, followed by a description and analysis of the fidelity to OD principles by these institutions.

#### 3.1.1. Implementation of OD in Germany

Table 1 provides an overview of the structural characteristics of all the participating institutions: in all cases, the majority of OD teams were outpatient service providers under public/non-profit ownership. Half of the teams offer OD under cross-sectoral model contract funding conditions. Among them, the hospital-based services, which are mostly responsible for acute treatment in Germany, offer OD exclusively under these model conditions. Moreover, in 47% of participating facilities, funding innovations, such as cross-sectoral model contracts described in the introduction, were the triggering factor to start the OD implementation process, which again highlights the importance of structural preconditions for this to occur.

**Table 1:** Characteristics of OD providing facilities/teams in Germany (*N* = 38).

N		38	
**Service structure**		***n***	**%**
Sector	Public/nonprofit	29	76
	Private	9	24
Service focus	Outpatient	22	58
	Inpatient	0	0
	Both	16	42
Service integration	Stand-alone (versus integrated service)	24	63
Treatment responsibility for catchment area		24	63
Open 24 h		14	37
Legal framework (funding) for OD^1^	Cross-sectoral model contracts (§64b, § 140a)^2^	20	53
	Standard care contract	19	50
**OD service structure**			
Trained staff	(Almost) all	17	45
	Majority	9	24
	< 50%	17	45
Reasons for implementation^1^	Dissatisfaction with current service	19	50
	Strengthening of caregiver support	18	47
	Change of funding model	17	45
	Practical experience in OD	11	29
	Strengthening the psychotherapeutic orientation	11	29
	Improving cooperation among colleagues	9	24
Clients’ age^2^	<18	8	21
	18-65	38	100
	>65	27	71

1 multiple answers possible.

2 “model contracts” via “model projects” according to §64b, SGB V and “integrated care contracts” according to §140a, SGB V.

By the time of the survey, the OD services in the participating facilities had existed for an average of 6.1 years (SD = 5.1), demonstrating rather recent implementation. The time between the first preparatory work and the first network meeting was 1 year on the average (SD = 1.2), showing a rather short implementation duration. In most cases, the first impulse for implementation came from the mid-level (30.9%) or senior management level (54.8%). In a good 45% of the facilities, almost the entire staff had been trained, but in another 45%, less than half of the staff received OD-training. The majority of the OD-practicing employees were social workers and nursing staff and in 66% of the facilities, peer support workers were members of the OD team.

#### 3.1.2. Fidelity to OD principles

**Figure 1:**
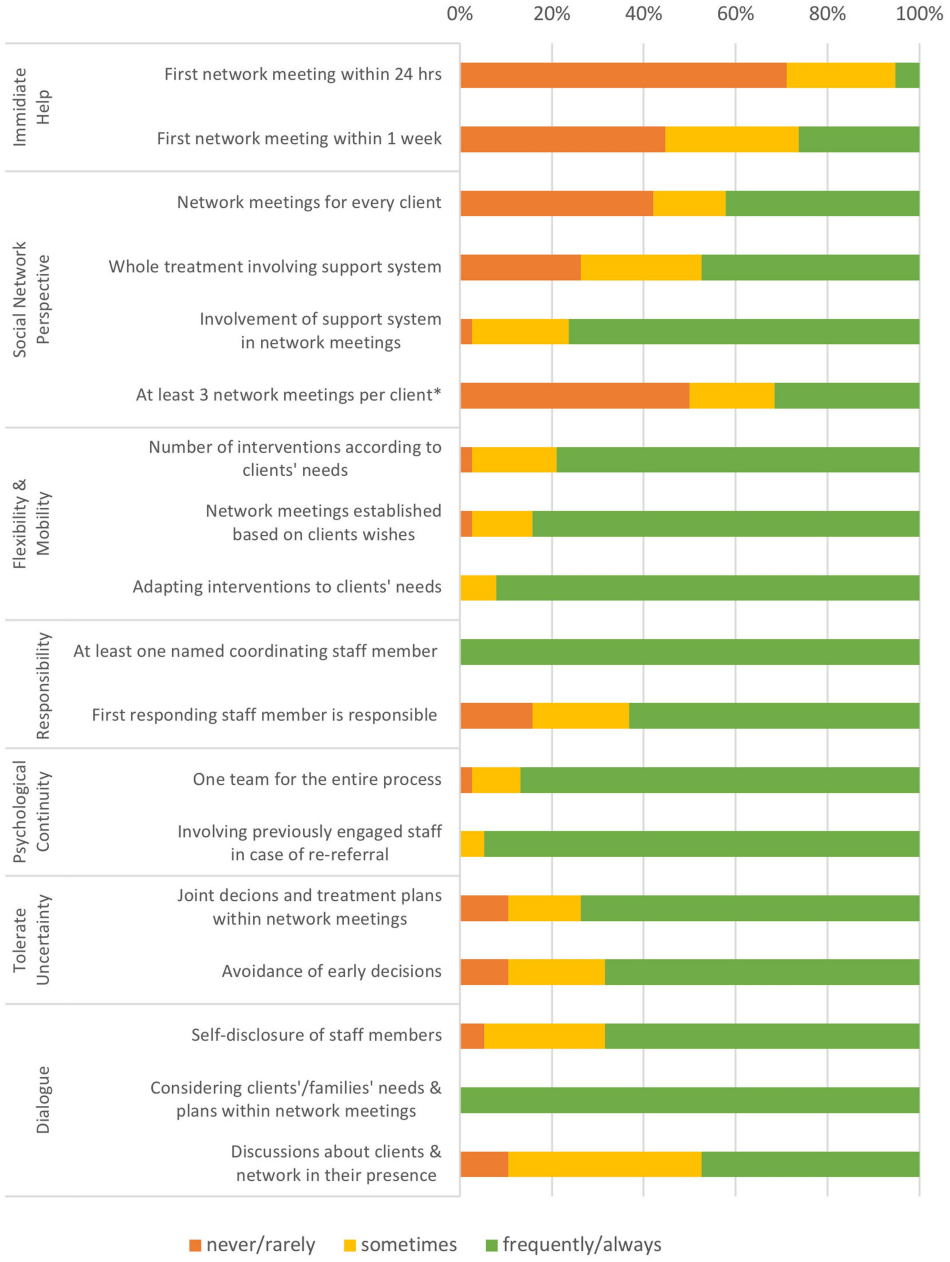
Extent of implementation of the OD principles across all participating teams, self-assessed with the HOPEnDIALOGUE survey. *additional item for the German examination.

Figure 1 shows the self-assessed extent of implementation of the OD principles across all participating teams, as assessed using the operationalization of the HOPEnDIALOGUE survey (except for one additional item). 71.1% of the facilities reported that they are rarely or never able to offer a network meeting within the first 24 h, and 44.7% declared that such an offer was not possible within the first week of treatment. 42.1% of the teams do not regularly offer network meetings for all their clients and 26.3% do not provide an entire treatment process together with the clients and their networks. Finally, about 50% rarely or never offer their clients more than two network meetings in total, drawing on an item that had been added to the survey only at the German research sites.

On the other hand, when network meetings do take place, the majority of the teams design the meetings according to the needs of the participants, both in terms of content (100%) and location (84.2%). The same applies to the amount (78.9%) and type (92.1%) of interventions regarding the entire treatment beyond network meetings. In addition, most of the teams appoint one dedicated staff member to coordinate the whole treatment process for clients (100%) and form a consistent team for the entire treatment process (86.8%). Regarding decision-making processes, 68.4% of the teams try to prevent early decisions on the treatment plans and 47.4% tend to discuss them openly during network meetings.

With regard to a potential interrelation with the financial and structural framework conditions, there were almost no statistically significant differences in the estimated fidelity to the various items on OD principles between facilities with different funding conditions or between inpatient and outpatient services. The only difference was that facilities with cross-sectoral model funding under §64b or §140a SGB V, were able to offer their clients 3 or more network meetings, i.e., significantly more often than those without (U = 148.5, *Z* = −2.298, *p* < 0.02). The same applies to teams without the treatment responsibility for a particular catchment area compared to teams with such responsibility (U = 142.5, *Z* = −2.201, *p* < 0.028).

### 3.2. Expert interview data

To understand the obstacles and challenges of OD implementation in more depth, expert interviews were conducted with 16 clinicians working in OD services in different contexts. The mean age was 49.56 years (SD = 10.85), and participants had an average work experience of 21.06 years (SD = 10.89). Moreover, they differed in their occupational groups and working contexts (see Table 2).

To facilitate a comparison between the results of the survey and the interviews, the obstacles to OD fidelity are presented first followed by a more comprehensive description of implementation challenges that are summarized under three levels of complex systems (macro-, meso- and micro-level) for the sake of clarity.

#### 3.2.1. Obstacles to implementation Fidelity

Different levels of fidelity to OD principles were also evident in expert interviews. Many participants reported that their teams offer flexible, continuous and mobile treatment, but this applies rarely to network meetings or “emergency-related” support:

"There are people who we see for half an hour a week, and there are people that we visit for two to three hours, three times a week. But I honestly wouldn't say that is because of Open Dialogue, but because of the way we work in our outpatient setting." (participant 9, social worker)

"[…] we ask [the patients] where they want the network meetings to take place, and most of the time it's at home. Sometimes they prefer to come to the hospital. And we've also held network meetings in a café or something." (participant 4, psychiatrist)

Providing immediate help was seen in most cases as impossible, as the majority of the facilities offer no crisis services whatsoever. Instead, immediate or initial contact is made possible within the first 24 h *via* telephone or emergency hotlines that had been set up for this purpose. In a few teams, where meeting personally within 24 h was considered possible, this mainly concerned individuals, as network meetings would hardly be possible to organize within such a short time frame:

“We don't provide immediate help, that's not what we offer. I have already considered offering network talks in crisis situations. But we haven't managed it yet.” (participant 3, social worker)

"Immediate help in case of a crisis, that's already quite a juggling act [...] especially when our whole day is already filled with appointments." (participant 5, psychologist)

Even beyond situations where immediate help would be needed, many interviewees regarded regular network meetings as “impossible,” despite the fact that network orientation was seen by all interviewees as a significant part of their care work:

"We strive for network meetings, but realization is not always possible or desired." (participant 14, psychologist)

#### 3.2.2. Challenges at the level of the mental health care system and policy (macro-level)

At the level of the health care system, a number of “structural problems” were described that make implementation of OD difficult. In particular, the fragmented nature of the German mental health care system results in a situation of numerous and often blurred treatment responsibilities:

“[…] you have to be careful, if the patient has an outpatient neurologist, an outpatient psychotherapist, then it is again difficult [to ensure] that there is no overlap in care.” (participant 11, psychiatrist)

“[If] someone calls [...] I'm not allowed to do anything. I always have to send them to the local health authority, so that they come to us after this control round, so to speak, and only then I am allowed to [start treatment]. So, this whole crisis thing only works from the moment the client is already part of our system.” (participant 12, nurse)

As a result, reacting quickly in the case of first contact is often very difficult or impossible, a situation that is also related to unclear or non-existent funding:

“[...] having a team [...] come within 24 hours […] is not possible due to the health care systems and the contracts we have.” (participant 2, social worker)

“A quick response is not possible as we must first clarify the health insurance’s coverage of the treatment.” (participant 2, social worker)

Moreover, some financial frameworks only allow for a quite restricted range of options, instead of providing for needs-oriented, continuous, and flexible support:

“These two staff members will stay only as long as the insurance contract lasts. That is only possible for three years.” (participant 2, social worker)

“The contracts we have with the health insurance companies basically determine how much money we get per client per year. They change the amounts again and again, which has lowered payments, and made eligibility criteria higher for clients. As a result. we were supposed to provide the same care with less and less money, which made it difficult to say: “We'll […] still work in pairs, we'll still go to people's homes, we'll still have network meetings.” That has definitely made it more difficult for us.” (participant 2, social worker)

More concretely, these inadequate financial conditions result in a lack of resources in time and staff to provide OD sufficiently with its different aspects. As the German health care system provides resources only for the treatment of individuals, involving his or her social network is not covered by insurance. This also applies to the additional time needed to organize network meetings and other administrative tasks related to implementation of the OD approach:

“We do not meet regularly in the patient’s home environment. We don't have the time for that. We meet too many people during the day for that.” (participant 5, psychologist)

“[…] we only have limited time resources, which […] makes it not so easy to design a long-term treatment process.” (participant 7, nurse)

“You end up faced with limitations in terms of personnel, of course. You can't even manage to have a [...] reflective team of several people there. [...]” (participant 5, psychologist)

[The] “organization is an obstacle [...] we have relatively little time for the clients [...] there is simply a lot of administrative stuff to do around it.” (participant 12, nurse)

Lastly, difficulties with implementing OD were mentioned that are the result of the health care system’s focus on outcomes and solutions, instead of processes:

“This goal-orientation, which is prevalent everywhere, means that we have to formulate goals and then work furiously to achieve them; this sometimes makes it difficult to remain in the here and now.” (participant 7, nurse)

#### 3.2.3. Institutional obstacles (meso-level)

Structural deficiencies were also reported at the level of the individual facilities. Many interviewees expressed their wish for a more substantial structural integration of OD work across their various facilities. In their opinion, this would only be possible if other treatment offers are discontinued and a change of attitude happens:

“We had to give up some of the old ward structures. Simply to be able to react more flexibly. Network meetings are quite time consuming and in order to guarantee we have enough time, we have reduced the number of treatment groups.” (participant 4, psychiatrist)

“What makes it more difficult are the traditionally designed structures. […] there is still little idea that psychiatry can also be done differently.” (participant 16, psychologist)

Accordingly, some interviewees wished for more understanding of the OD method within the entire institution. They especially stressed the importance of a sufficient number of trained employees to ensure that implementation did not fall on too few shoulders:

“It needs a critical mass, which is what I said earlier. It is always difficult when staff members are alone in the facilities […]. At the same time, it is important to bring along the others who have not done the training, but who need to know what is going on, so that they do not have the feeling that this whole story is passing them by.” (participant 1, psychologist)

In this aspect in mind, the high turnover of employees was deemed problematic, especially in hospital environments, where staff continuity is a problem anyway:

“What continues to be a fundamental problem, of course, is staffing. In general, […] there is simply a lot of change at all levels. A lot of inconstancy. Especially in the nursing sector, due to shift work, part-time staff, absenteeism. That is certainly the biggest challenge to create a minimum amount of continuity.” (participant 2, social worker)

The lack of integration within an institution was also perceived to be related to a lack of support from the management level. A greater commitment and understanding coming from this level was desired in order to facilitate the OD implementation process. Participants objected to a primarily economic interest on the part of management, which was reflected in job cuts, among other things:

“uh, they have always been struggling and, in my opinion, this was due to the [...] inadequate support by management level.” (participant 7, nurse)

“It only works when the leaders are on board. They don't have to undergo the training themselves, [but] they must have knowledge of Open Dialogue [and] there has to be a commitment. [...] Part of the implementation must be top-down.” (participant 1, psychologist)

“There is always the threat of job reduction. I also have the impression that the hospital management […] does not look at what patients need, but only at what they can make money with. […] And of course, Open Dialogue suffers from this, because it is a bit more personal and time-consuming than just prescribing medication.” (participant 11, psychiatrist)

In the view of many interview participants, cooperation with providers of other mental health and social services is also impaired by the lack of financial capacities to include treatment facilitators from outside of the institution:

“When we organize a network meeting, we ask the client's psychiatrist to leave her practice and come to our facility and sit down for two/three hours. On a financial basis, that is simply impossible.” (participant 1, psychologist)

Moreover, too little dissemination of OD in other services and a lack of communication among practitioners within the region often lead to low visibility and understanding of this approach:

“It has certainly made it more difficult that there is no networking with other institutions that also do this.” (participant 3, social worker)

“What we would need, is a utopia: broader implementation in the regional care system, so that others know what we are talking about when we invite people to a network meeting.” (participant 1, psychologist)

#### 3.2.4. Individuals’ resistances and reservations (micro- level)

At the day-to-day work level, participants primarily pointed to resistances or reservations about the OD approach as a further obstacle. In some interviews, a degree of “innovation fatigue” due to the constant introduction of new concepts in psychiatry was mentioned to explain this:

“New things are always coming onto the market and then they are hyped to the point of no return, and at some point, they fall apart and then the next one comes along. That's rather counterproductive and makes many people skeptical.” (participant 7, nurse)

“I had a conversation with a manager from another facility. He told me: ‘I have supported so many supposedly promising projects over the last years and invested so much time in them, and it has often come to nothing - I don't want to do it anymore’” (participant 7, nurse)

For OD novices, the introduction of this approach means being diverted from previously accustomed practices, like a one-to-one and solution-oriented approach. Working in treatment tandems also initially means additional work, raising questions of its benefits for some of them:

“If you already have this attitude: "I don't have the solution", then it's not difficult to accept OD as a treatment form. Yet, for new colleagues, or if you come from a completely different conceptual background, then this may be difficult and a huge challenge.” (participant 2, social worker)

“Some staff members simply don’t have the mindset to work according to these principles and find […] working alone better.” (participant 13, social worker)

“[…] they often find it too complicated to work in a tandem. […] asking the question: first of all, it requires a lot of time and what is the benefit?” (participant 1, psychologist)

Moreover, the shift to a dialogical way of dealing with different opinions and decisions may challenge previous, long-existing attitudes and behaviors. For example, the change of attitude from “talking about” to “talking with” was reported to cause great difficulties for many colleagues:

“The temptation to return to old behavior patterns and follow an idea in your own head and to pursue that, instead of asking in a more open way, is sometimes difficult.” (participant 13, social worker)

“[…] due to reasons of time and probably also habit, I exchange information with the physician in charge far too often, without having the patient next to me.” (participant 6, psychiatrist)

Going into more depth, handling of responsibility was perceived to be a challenge. On the one hand, it is difficult for many staff members to leave the responsibility for some decisions up to the network. On the other hand, the OD approach requires taking on more responsibility than was previously the case or even desired:

“[…] when do we want to meet again next time? And when the patient answered, "in about three months or so", this seriously perturbed certain staff members who had great difficulty to accept not having contact for so long, and they first had to develop trust in the patient's resources.” (participant 7, nurse)

“And then there are people who feel more secure in their work in an inpatient context, because there are still colleagues to back them up, because they know they can hand over [the responsibility] and there is someone there to take it on.” (participant 5, psychologist)

In extreme cases, these reservations played out as quite frankly expressed utterances of professional competition among colleagues from different professions or working for other service providers:

“We had extreme problems at the beginning, there was really a lot of competition. Maybe because many doctors are used to being the practitioner and deciding about the treatment of the person of concern. That was really difficult at the beginning. There were doctors who said that if the patient let us accompany him, then they would stop treating him or her as a patient.” (participant 2, social worker)

“Resentment always, definitely. Most of all, by the way, with the medical profession, when you suddenly start wanting to have your say, so to speak.” (participant 12, nurse)

“Others have said, "We've always kind of done it this way. We don't need to deal with that." Or some have said: "We can't do that".”

Given these reservations, it is interesting to note that some of the interviewees found that rolling out OD in too short of a timeframe or with an overly radical desire for change represented additional obstacles promoting reservations from the outside:

“At the beginning, I thought: we'll change to network meetings and not allow for any other forms of treatment anymore. That is, of course, nonsense. That can lead to people saying at some point: "We're not going to do anything anymore. Because the goal is too big. [...] you can also only start with single aspects".” (participant 3, social worker)

“If you didn't call it Open Dialogue all the time, if you didn't sell it as the miracle concept from Finland, so to say, it would not scare people off so much [...]” (participant 16, psychologist)

Finally, it appeared in various interviews that a high level of individual commitment is required for successful OD implementation. This factor, in addition to the many obstacles described above led to frustration among some participants:

“But this attitude is just not there yet. It's hard, you have to repeat it again and again and bring it in. It gets tiring over time if it always comes from me and there is little initiative from the others to educate themselves and try something out.” (participant 11, psychiatrist)

## 4. Discussion

This article is the first to examine the spread and extent of OD implementation in Germany, together with the challenges and obstacles involved. Overall, the results of the survey demonstrated that a large number of facilities within various care contexts have implemented certain elements of OD. At the same time, the full set of OD principles was not simple to implement, which especially applies to the principles “immediate help in case of crisis” and “network perspective,” which are currently only implemented on a regular basis by a few teams in Germany. Instead, mobile, flexible, and continuous support is implemented more consistently over time, as shown by the survey results, whereas the expert interviews demonstrated challenges in this regard as well.

Overall, the grade of implementation of OD structural principles was far lower than implementation of therapeutic principles. Presumably, this was mainly due to contextual contingencies, as expert interviews revealed that the teams investigated did their best to implement the OD principles as fully as possible under the conditions of their respective service structure and the German health care system. These efforts resulted in the achievement that, in international comparison, Germany has the highest number of teams practicing OD to be found, as described in the global HOPEnDIALOGUE survey (Pocobello et al., 2022). On the other hand, the mental health structures in which the German OD-teams operate are significantly more stand-alone services than the OD-teams of other countries and report significantly less often to provide the OD principles: “Immediate help,” “Social Network,” Tolerate Uncertainty” and “Dialogue” (*ibid*). Thus, the high number of providing services must be treated with caution, as discussed below.

### 4.1. Contextualization of findings in relation to the German mental health care system

Of all participating teams, 42.1% do not offer network meetings on a regular basis for every client and only about 50% offer not more than two network meetings in total for every client. This situation is even more regrettable as network meetings are the central instrument of the OD approach. Likewise, immediate help with a network meeting within 24 h appears to be equally important to achieve desired OD outcomes. Therefore, the overall lack of implementation of this OD principle in Germany for 71.1% of the facilities surveyed may be considered rather problematic.

At the same time, there was a very simple explanation for these results, given the structural contexts of most of the participating services: Only 37% were open 24 h a day and 58% were outpatient services - which in Germany are usually not responsible for crisis care. In more detail, the contract terms of the integrated care services according to §140a (see above) required that clients were first contacted by their health insurances and had to enroll themselves after a further clarification process. This enrollment process was clearly too long for situations, in which immediate help to people in crises was needed. Thus, the included clients usually were not in a crisis state at the beginning of OD support. To give another example, practicing OD in the context of residential care usually meant that therapeutic relationships had been existing for longer times, sometimes years, so that the search for facilitators still unknown to the network often took some time, which is usually not available in the case of an acute crisis. In these cases, further, a social network first had to be activated, which again took time, before a network meeting could take place.

On the other hand, it should not be underestimated that a large part of the teams attempted to provide a needs-oriented and flexible support. The majority stated that the content (100%) and location (84.2%) of network meetings as well as the type (92.1%) and extent (78.9%) of further interventions were aligned as closely as possible to the needs of the clients. Conversely, in our view, this finding also confirms the structural dependence of the implementation possibilities of OD in Germany: Since these elements can be more easily adapted to existing structures and concepts of the German mental health care system, flexibility and mobility were usually indicated to be part of the routine care of the participating centers.

Altogether, the fragmented und institutionalized conditions of the German health care system indeed seem to hinder a comprehensive implementation of the OD principles. Accordingly, as mentioned before, the extent of implementation of OD in different countries and contexts is highly dependent, among other things, on the health care system (Buus et al., 2021). In its original development, the full implementation of the OD approach requires a distinct set of structural changes over several years (Haarakangas et al., 2007). In contrast, in our study, the average time between the first preparatory measure and the provision of the first network meeting was 1 year. And often, the implementation was not supported by the entire institution or even the leadership, as was clearly evident in the interviews. Thus, without a sufficient structural basis, a high number of OD teams in Germany must be presumed to depend largely on individual commitment, which was also addressed in some of the interviews. Such a bottom-up implementation of OD may lead to gradual adaptation of clinical practice but cannot provide a sufficient basis for a full change of existing structures and practices, as discussed earlier (Buus et al., 2021). Thus, it is understandable that the need for top-down implementation was emphasized in some interviews.

#### 4.1.1 Legal and financial constraints

Regarding the legal conditions, half of the German OD teams were providing services under cross-sectoral model contracts according to §64b or §140a treatment conditions at the time of the survey. The results also revealed that the majority of OD implementation processes were preceded by introduction of one of these funding conditions in almost half of the survey cases, either triggering it or making it possible in the first place. Moreover, a detailed analysis demonstrated that the item “more than three network meetings per client” was shown to be significantly associated only to those institutions using cross-sectoral model funding, indicating a greater scope for a need-adapted support within these contracts. Thus, as expected, cross-sectoral model conditions do seem to have opened possibilities for implementing OD and a more flexible form of care. At the same time, as described before, those contracts include restrictions in terms of accessibility and duration available for the treatments offered and thus are not suited to secure stable and continuous treatment, as recurrently emphasized in the expert interviews of our study.

As a particularly dramatic example, an impressive network of OD services had been built up in Berlin and elsewhere (Mueller-Stierlin et al., 2017), in which multi-disciplinary teams offered crisis and assertive forms of home treatment to prevent hospitalizations on the basis of an integrated care contract (according to §140a SGB V). Yet the contract was signed only by one large and some singular regional insurance companies, denying access to patients of the roughly 140 other insurance companies. Furthermore, in 2021, most of the contracts were canceled, after years of gradual cutbacks with the result that the providers participating were forced to stop their OD services, after having acquired valuable (and expensive) expertise for years and mostly without being able to offer adequate alternatives to their clients. Thus, OD projects under cross-sectoral model conditions (§64b and §140a, SGB V) usually have a limited funding duration even if their results would often justify a transfer to standard care.

In our view, the wider context for this situation is the increasing economization of the (German) (mental) health care system, leading to a significant cutback of jobs, as well as the use of selective contracts covering expenses only for brief treatment periods or short-term interventions, as mentioned frequently in most of the interviews. Fittingly, inadequate funding structures and lack of resources are a common obstacle to the adequate implementation of OD approaches (Buus et al., 2021), which were also mentioned several times in our expert interviews. This trend seems to be spreading ever further in Germany, pointing to the lack of the political will to change it – or as one of our interview partners framed it: “OD must be politically desired. But this kind of work has no lobbies behind it. They [cannot] make any profit out of it. [...] Because our capitalist system has no interest in it.” (participant 1, psychologist). Inversely, health economic analyzes indicate that most of Germany’s financial resources are spent for inpatient treatment and outpatient drug prescriptions while only a small part of the mental health care budget is spent on outpatient services (Heider et al., 2009; Salize et al., 2009). Thus, it could be expected that the German mental healthcare system could be significantly improved by shifting resources from inpatient to outpatient care. Yet, legal regulations applicable to health care financing, as well as organizational issues, represent major barriers against this shift from inpatient to outpatient as well as from medical to psychosocial services (Bauer et al., 2016).

### 4.2. Is it structure – Or is it attitude?

At the same time, both the survey and interview data made clear that certain OD elements were implemented despite the limiting structural conditions under different funding contracts, demonstrating that an extensive restructuring of mental health care conditions may not be a *sine qua non* for the implementation of *all* principles. Conversely, even if a team was funded according to the more flexible cross-sectoral model contracts, this did not necessarily guarantee that a great extent of implementation of (all) OD principles had been achieved, indicating that there must be other reasons than the health care context to explain for this shortcoming.

The reasons for this finding relate to obstacles on different levels. For example, expert interviews revealed various obstacles both on a meso- and micro-level, such as a lack of support from the senior or executive management. Thus, as shown in results from other studies, it may be argued that the implementation of OD inevitably leads to challenges and can only succeed through an adaptive and committed leadership (Lennon et al., 2022) and organizational change management processes (Buus et al., 2021).

On the other hand, in terms of implementation, not only the structural principles’ implementation seemed to depend on an upstream change of the health care context, but also certain therapeutic principles may depend on structural conditions for their (full) implementation. For instance, “asking open questions” or “tolerance of uncertainty” (instead of seeking quick solutions) may require more time, which in the end means resources. This may explain why only about two-thirds of the institutions participating in the survey try to prevent early decisions or tend to discuss them openly during network meetings.

Other therapeutic principles seem to be less structurally dependent, but they primarily require a change of attitude to be implemented: for example, discussions about the clients and networks in their presence were reported by only 47.4% of the participants to be applied at their institution regularly. Supported by the interview data and other studies (Dawson et al., 2019; Tribe et al., 2019), this leads to the hypothesis that in addition to the lack of adequate structural conditions, barriers for implementation can also be found in the attitudes of individual staff members or an institutional culture as a whole, caused by personal doubts or resistances, when an OD principle challenges previous treatment routines or approaches.

### 4.3. Why do we need fidelity?

The significant impact of attitude and the required change of culture mentioned leads to the question of what actually constitutes the OD approach and at what point an implementation can no longer qualify as “Open Dialogue.” It has been argued that clear, transparent, and accepted criteria of fidelity are important to ensure and monitor sufficient implementation (Waters et al., 2021). If and how the OD approach is implemented or not, should not depend on personal decisions or tastes but requires clarity, consistency, and careful implementation of principles to ensure good quality care for clients and their networks, as well as to facilitate further research on the outcomes of OD.

To pursue this goal, a set of clear and communicable criteria is needed to analyze the extent to which this approach has been delivered and the quality level (Waters et al., 2021). Such a set is also needed for various reasons: first, to allow for communication on this topic among members of the OD community and externally, second to facilitate the transferability and translation of this approach in various contexts, and third to reduce harmful processes with definitional power regarding the “real nature of OD” (Von Peter et al., 2022).

At the same time, finding adequate fidelity criteria for the OD approach looks like quite a complex task. The inbuilt principles of openness, need-adaption and flexibility make distinct definitions challenging (Waters et al., 2021) without devaluing these core elements. Thus, there could be a risk of ruining the very foundations of the approach if fidelity criteria are too strict or normative, thereby potentially obstructing helpful variances of the OD approach for users and caregivers. It is useful to present one example of these variances. As a creative response to the structural constraints limiting full implementation of the OD approach in Germany, a variety of modified versions have been developed. For example, the so-called treatment conference has been developed on an acute care unit to replace the traditional, rather top-down senior physician’s rounds in such a way that allowed for a connection and transition to the newly introduced dialogical network meetings (Aderhold et al., 2010). These conferences are applied in some hospital departments today allowing for more dialogue and reflections among the team in the presence of the patient to provide for feedback to the patients of concern and request their response with or without additional practice of network meetings which is justified with lack of time.

These and other, certainly worldwide existing variants of the OD approach would not qualify as OD or would likely not have been developed in the first place, if too narrow of a focus on a definite set of fidelity criteria takes precedence over the nature or quality of the OD implementation process. Thus, fidelity criteria should make it possible to talk more clearly about the framework conditions, processes, and extent of implementation, but should not restrict the range of possible OD practices.

Even more importantly, the (non-)implementation of any of the OD practices or respect of fidelity criteria cannot measure users’ and carers’ experiences of an OD service. Given the current lack of adequate studies that confirm the causalities between OD fidelity and outcomes, we must be cautious about drawing overly narrow conclusions on this issue. Hopefully, the ODDESSI trial will shed some light on this question (Pilling et al., 2022), but even if it does, this will not make it possible to define these associations authoritatively in a particular clinical situation. In this respect, a plurality of “evidence” could be useful in our opinion, challenging the evidence-based medicine hierarchy of knowledge and bringing to the fore the highly important narrative evidence that has been produced since the beginning of OD development, largely contributing to its current shape and effectiveness.

It remains to be seen if better terminology to differentiate and communicate the differences in implementation and variances of the OD approach is needed for these purposes. While collaborating on this publication, we experimented with various terms, such as “OD-oriented” or “-inspired” services, or “dialogical networking,” also inspired by similar discussions in relation to Soteria services that use a corresponding fidelity scale to clearly differentiate grades of implementation. However, we decided not to use these terms in the end here, as each of them would require a more extensive discussion, with other authors in addition to our research team, to determine what counts as “full” or “minor” OD implementation.

### 4.4. Limitations

The limitations of the survey relate to the questions used, as they were not based on a validated questionnaire, thus limiting the accuracy of the results. It should also be noted that the survey data collected were exclusively self-reported, which also affects the validity of the results. Moreover, the responses for each team were always gathered by one person and it was not possible to check whether the responses were coordinated with all of their team members. In this respect, the survey results should be understood as an approximation and not as a precise picture of the OD care landscape in Germany. On the other hand, a distortion of the data in the sense of social desirability would, in our view, be expected above all in the levels of implementation of the fidelity criteria. It was precisely here that the results were particularly sobering.

The limitations of our qualitative study first came from the limited number of expert interviews. As a result, this aspect of the study suffered from the shortcoming that it did not involve one representative of each participating institution of the survey study. Due to the restriction of resources, it was not possible to include a larger number of interview partners. However, during data analysis, we recognized a sufficient saturation of the material, with similar themes repeating again and again over the course of all interviews, making the approximations based on comprehensive results. This article focuses on the topic of implementation challenges and obstacles, rather than a discussion of possible solutions for them, including those mentioned in interviews. The goal of this sub-study, however, was to understand these challenges in depth, which might not have been possible if also discussing the possible ways out.

## 5. Conclusion

Despite various structural and other barriers, a large number of teams, working in the field of psychosocial and psychiatric care in Germany, apply OD related elements in their treatment approach. As shown, OD is not implemented to its full extent in each of the institutions surveyed. This has led to the suggestion to start a broad discussion among OD researchers, practitioners and clients to develop a more refined terminology to define and communicate variants of OD implementation, involving terms such as “OD-oriented” or “OD-inspired” services. This article has made it clear that more extensive implementation of the OD approach in Germany, as maybe elsewhere, is prevented mainly by the conditions of the health care system. This is even more worrisome, as community-based, flexible, and needs-oriented forms of psychosocial care are strongly recommended by many guidelines today, regardless of the OD approach (Gühne et al., 2018; WHO, 2021).

During the production of this article, the authors, as OD trainers and practitioners who are attached to this approach, frequently oscillated between appreciation for what the German teams had made possible despite the prevailing adverse health care conditions and disillusionment seeing how in certain cases, OD principles were implemented in a rudimentary way. Yet, these feelings of ambivalence may also apply to other health care sectors too, the implementation of the OD approach usually being dependent on significant adaptations of the health care context (Buus et al., 2021). Finally, this article was not designed to provide information on the outcomes of the services involved on the experiences of the users and caregivers benefiting from OD. These additional aspects must be covered *via* a subsequent study to record and analyze the effectiveness of OD services in Germany.

## Data availability statement

The raw data supporting the conclusions of this article will be made available by the authors, without undue reservation.

## Author contributions

KH co-developed the expert interview questionnaire, revised, and adapted the code tree, re-analyzed the survey data and wrote the article. MK developed the German adaptation of the cross-sectional survey, conducted the examination, and first analysis and co-developed the expert interview questionnaire. MB and HT developed the expert interview questionnaire, conducted, and evaluate the expert interviews. RP developed the questionnaire of the cross-sectional survey, helped with the analysis. YI helped with adaption, conduction, and analysis of the German survey. VA co-developed the expert interview questionnaire and the German adaption of the survey, helped with the recruitment for both survey and expert interviews, discussed the overall results. SP developed the expert interview questionnaire, evaluate the expert interviews, developed the German adaptation of the survey, discussed the overall results, and co-produced the article. All authors contributed to the article and approved the submitted version.

## Funding

Funded by the MHB publication fund supported by DFG.

## Conflict of interest

The authors declare that the research was conducted in the absence of any commercial or financial relationships that could be construed as a potential conflict of interest.

## Publisher’s note

All claims expressed in this article are solely those of the authors and do not necessarily represent those of their affiliated organizations, or those of the publisher, the editors and the reviewers. Any product that may be evaluated in this article, or claim that may be made by its manufacturer, is not guaranteed or endorsed by the publisher.

## Supplementary material

The Supplementary material for this article can be found online at: https://www.frontiersin.org/articles/10.3389/fpsyg.2022.1072719/full#supplementary-material

## References

[ref1] Aaltonen J. Seikkula J. Lehtinen K. (2011). The comprehensive open-dialogue approach in Western Lapland: I. the incidence of non-affective psychosis and prodromal states. Psychos 3, 179–191. doi: 10.1080/17522439.2011.601750

[ref2] Aderhold V. Borst U. (2016). “Stimmenhören Lernen”– Qualifizierung für systemische Arbeiten in der psychiatrischen Grundversorgung. Familiendynamik 41, 34–43.

[ref3] Aderhold V. Gottwalz-Itten E. Haßlöwer H. (2010). Die Behandlungskonferenz–Dialog, reflexion und transparenz. PPH 16, 142–152. doi: 10.1055/s-0030-1254513

[ref4] Bauer E. Kleine-Budde K. Stegbauer C. Kaufmann-Kolle P. Goetz K. Bestmann B.. (2016). Structures and processes necessary for providing effective home treatment to severely mentally ill persons: a naturalistic study. BMC Psychiatry 16:242. doi: 10.1186/s12888-016-0945-z, PMID: 27422014 PMC4946100

[ref5] Bergstrøm T. Alakare B. Aaltonen J. Mäki P. Köngäs-Saviaro P. Taskila J. J.. (2017). The long-term use of psychiatric services within the open dialogue treatment system after first-episode psychosis. Psychos 9, 310–321. doi: 10.1080/17522439.2017.1344295

[ref6] Bergström T. Seikkula J. Alakare B. Mäki P. Köngäs-Saviaro P. Taskila J. J.. (2018). The family-oriented open dialogue approach in the treatment of first-episode psychosis: nineteen–year outcomes. Psychiatry Res. Commun. 270, 168–175. doi: 10.1016/j.psychres.2018.09.039, PMID: 30253321

[ref7] Bramesfeld A. Ungewitter C. Böttger D. Jurdi J. E. Losert C. Kilian R. (2012). What promotes and inhibits cooperation in mental health care across disciplines, services and service sectors? A qualitative study. Epidemiol. Psychol. Sci. 21, 63–72. doi: 10.1017/s2045796011000539, PMID: 22670414

[ref8] Brooks H. Pilgrim D. Rogers A. (2011). Innovation in mental health services: what are the key components of success? Implement. Sci. 6:120. doi: 10.1186/1748-5908-6-120, PMID: 22029930 PMC3214129

[ref9] Buus N. Ong B. Einboden R. Lennon E. Mikes-Liu K. Mayers S.. (2021). Implementing open dialogue approaches: a scoping review. Fam. Process 60, 1117–1133. doi: 10.1111/famp.12695, PMID: 34322874

[ref10] Dawson L. River J. McCloughen A. Buus N. (2019). ‘Should it fit? Yes. Does it fit? No’: exploring the organisational processes of introducing a recovery-oriented approach to mental health in Australian private health care. Heal 25, 376–394. doi: 10.1177/1363459319889107, PMID: 31773989

[ref11] Gühne U. Weinmann S. Riedel-Heller S. G. Becker T. (2018). “S3-Leitlinie Psychosoziale Therapien bei schweren psychischen Erkrankungen: S3-Praxisleitlinien in Psychiatrie und Psychotherapie [German]” in Herausgebende Fachgesellschaft: DGPPN (Berlin: Springer)

[ref12] Haarakangas K. Seikkula J. Alakare B. Aaltonen J. (2007). “Open dialogue: an approach to psychotherapeutic treatment of psychosis in northern Finland” in Collaborative Therapy. eds. Anderson H. Gehart D. (London: Routledge)

[ref13] Heider D. Bernert S. König H.-H. Matschinger H. Hogh T. Brugha T. S.. (2009). Direct medical mental health care costs of schizophrenia in France, Germany and the United Kingdom–findings from the European schizophrenia cohort (euro SC). Eur. Psychiatry 24, 216–224. doi: 10.1016/j.eurpsy.2008.12.013 19328658

[ref14] Helfferich C. (2011). Die Qualität qualitativer Daten: Manual für die Durchführung qualitativer Interviews. Wiesbaden: VS Verlag für Sozialwissenschaften/Springer.

[ref15] Lennon E. Hopkins L. Einboden R. McCloughen A. Dawson L. Buus N. (2022). Organizational change in complex systems: organizational and leadership factors in the introduction of open dialogue to mental health care services. Commun. Ment. Hlt. J., 1–10. doi: 10.1007/s10597-022-00984-0 [Epub ahead of print], PMID: 35585467 PMC9813114

[ref16] Mueller-Stierlin A. S. Helmbrecht M. J. Herder K. Prinz S. Rosenfeld N. Walendzik J.. (2017). Does one size really fit all? The effectiveness of a non-diagnosis-specific integrated mental health care program in Germany in a prospective, parallel-group controlled multi-Centre trial. BMC Psychiatry 17:283. doi: 10.1186/s12888-017-1441-9, PMID: 28764729 PMC5539984

[ref17] Peter S. Von Ignatyev Y. Johne J. Indefrey S. Kankaya O. A. Rehr B.. (2019). Evaluation of flexible and integrative psychiatric treatment models in Germany—a mixed-method patient and staff-oriented exploratory study. Front. Psych. 9:785. doi: 10.3389/fpsyt.2018.00785, PMID: 30723433 PMC6349706

[ref18] Pilling S. Clarke K. Parker G. James K. Landau S. Weaver T.. (2022). Open dialogue compared to treatment as usual for adults experiencing a mental health crisis: protocol for the ODDESSI multi-site cluster randomised controlled trial. Contemp. Clin. Trials 113:106664. doi: 10.1016/j.cct.2021.106664, PMID: 34958932 PMC8844585

[ref19] Pocobello R. El Sehity T. J. Camilli F. Alvarez-Monjaras M. Bergstrøm T. Von Peter S.. (2022). A global survey on open dialogue. Front. Psychol. In submission 10.3389/fpsyg.2023.1241936 PMC10668593 38023059

[ref20] Proctor E. K. Landsverk J. Aarons G. Chambers D. Glisson C. Mittman B. (2009). Implementation research in mental health services: an emerging science with conceptual, methodological, and training challenges. Admin. Policy Ment. Health 36, 24–34. doi: 10.1007/s10488-008-0197-4, PMID: 19104929 PMC3808121

[ref21] Puschner B. Baumgartner I. Loos S. Völker K. Ramacher M. Sohla K.. (2012). Kosteneffektivität bedarfsorientierter Entlassungsplanung bei Menschen mit hoher Inanspruchnahme psychiatrischer Versorgung. Psychiatr. Prax. 39, 381–387. doi: 10.1055/s-0032-1327188, PMID: 23138329

[ref22] Salize H. J. McCabe R. Bullenkamp J. Hansson L. Lauber C. Martinez-Leal R.. (2009). Cost of treatment of schizophrenia in six European countries. Schizophr. Res. 111, 70–77. doi: 10.1016/j.schres.2009.03.027, PMID: 19401265

[ref23] Salize H. J. Rössler W. Becker T. (2007). Mental health care in Germany. Eur. Arch. Psy. Clin. N. 257, 92–103. doi: 10.1007/s00406-006-0696-9 17149540

[ref24] Schwarz J. Bechdolf A. Hirschmeier C. Hochwarter S. Holthoff-Detto V. Mühlensiepen F.. (2020). “Ich sehe es tatsächlich als Zwischenschritt”– eine qualitative Analyse der Implementierungsbedingungen und -hürden von Stationsäquivalenter Behandlung in Berlin und Brandenburg. Psychiatr. Prax. 48, 193–200. doi: 10.1055/a-1274-3662, PMID: 33307566

[ref25] Seikkula J. Aaltonen J. Alakare B. Haarakangas K. Keränen J. Lehtinen K. (2006). Five-year experience of first-episode nonaffective psychosis in open-dialogue approach: treatment principles, follow-up outcomes, and two case studies. Psychother. Res. 16, 214–228. doi: 10.1080/10503300500268490

[ref26] Seikkula J. Alakare B. Aaltonen J. (2001). Open dialogue in psychosis I: an introduction and case illustration. J. Constr. Psychol. 14, 247–265. doi: 10.1080/10720530125965

[ref27] Seikkula J. Alakare B. Aaltonen J. (2011). The comprehensive open-dialogue approach in Western Lapland: II. Long-term stability of acute psychosis outcomes in advanced community care. Psychos 3, 192–204. doi: 10.1080/17522439.2011.595819

[ref28] Seikkula J. Arnkil T. E. (2006). Dialogical Meetings in Social Networks. London: Routledge

[ref29] Søndergaard K. D. (2009). Innovating Mental Health care. A Configurative Case Study in Intangible, and Incoherent and Multiple Efforts. Aarhus: The Danish School of Education, Aarhus University

[ref30] Steinert U. Diekmann P. Meyn C. Frommhagen M. Aderhold V. Von Peter S. (2020). Neugier bringt uns weiter – oder warum wir lernen, immer wieder fragen zu wollen. Pph 26, 199–205. doi: 10.1055/a-1160-3129

[ref31] Tribe R. H. Freeman A. M. Livingstone S. Stott J. C. H. Pilling S. (2019). Open dialogue in the UK: qualitative study. Bjpsychol. Open 5:e49. doi: 10.1192/bjo.2019.38, PMID: 31189488 PMC6582213

[ref32] Von Peter S. Eissing K. Saliger K. (2022). Open dialogue as a cultural practice - critical perspectives on power obstacles while teaching and enabling OD in current psychiatry. Front. Psychol. accepted doi: 10.3389/fpsyg.2022.1063747 PMC9983634 36875543

[ref33] Von Peter S. Heumann K. Kuhlmann M. Aderhold V. (2020). Der Offene Dialog und seine Anwendung in Deutschland. Pph 26, 191–198. doi: 10.1055/a-1160-2847

[ref34] Von Peter S. Von Aderhold V. Cubellis L. Bergström T. Stastny P. Seikkula J.. (2019). Open dialogue as a human rights-aligned approach. Front. Psych. 10:387. doi: 10.3389/fpsyt.2019.00387, PMID: 31214063 PMC6555154

[ref35] Von Peter S. Von Bergstrøm T. Nenoff-Herchenbach I. Hopfenbeck M. S. Pocobello R. Aderhold V.. (2021). Dialogue as a response to the Psychiatrization of society? Potentials of the open dialogue approach. Front. Sociol. 6:806437. doi: 10.3389/fsoc.2021.806437, PMID: 35004940 PMC8727686

[ref36] Waters E. Ong B. Mikes-Liu K. McCloughen A. Rosen A. Mayers S.. (2021). Open dialogue, need-adapted mental health care, and implementation fidelity: a discussion paper. Int. J. Ment. Health Nutrit. 30, 811–816. doi: 10.1111/inm.12866, PMID: 33848029

[ref37] WHO (2021). Guidance on Community Mental Health Services: Promoting Person-centred and Rights-based Approaches. Geneva: World Health Organization.

