# Supplementary material for: Implementation of open dialogue in Germany: Efforts, challenges, and obstacles
Source: Front Psychol. 2023 Feb 9;13:1072719. doi: 10.3389/fpsyg.2022.1072719 (PMC9948650; doi:10.3389/fpsyg.2022.1072719)
Supplement: Supplementary file 1 [file Table_1.DOCX]

**Implementation of OD in different care contexts - challenges and opportunities**

What are the opportunities, barriers, and challenges in implementing OD due to the conditions of the mental health care system?

**Guideline expert interview**

| Nr. | Guiding question/narrative | Basic aspects | Specific questions/ suggestions for discussion |
| --- | --- | --- | --- |
| I | How is OD being implemented at your facility? | Implementation practice  Possibilities  Motivations / History | 1. Since when has OD existed in your unit? How did it come into exictence? 2. How permeated is your facility with OD/ is OD provided in a specialty unit? Or is your entire facility trained/"on board"? 3. How does networking work with other providers? Outpatient and inpatient? 4. What have you changed in your facility to make OD work? What had to be changed? 5. Which framework conditions have made it easier or more difficult to implement open dialogue in your facility? 6. Was there any resistance to the introduction of OD within your institution? For example, power struggles? 7. What are the advantages and disadvantages of using OD for your own work? Has OD changed the way of talking in the team, especially about patients? Has OD changed the focus/goal of treatment? |
| II | Which OD principles are applied in your practice? | Theory vs. practice  (discuss on the basis of the table with the key elements) | 1. Which of the OD principles shown can be applied well in their lived practice? Which ones are difficult? Where do you deviate from the principles and why? 2. How do you define a network meeting? What does it take to classify a treatment/conversation as a network meeting? 3. Is OD always applicable in your treatment situations? Or, in your view, are there groups/situations for which the approach or setting seems unsuitable? |
| III | What would have to change/ Under what conditions could OD be better implemented? | Desires and hopes | 1. What does it take to sustain OD work at your facility? What needs to be done to make OD work for you in the long term? 2. What framework conditions, e.g. on a structural or legal level, etc., would be needed to enable OD to be implemented even better in your company? 3. What experiences from your project could help other projects in the implementation of OD? 4. What do you think would make OD dissemination easier/more likely? 5. Is there anything else you would like to say/ ask? |
